# Milano Summer Particulate Matter (PM10) Triggers Lung Inflammation and Extra Pulmonary Adverse Events in Mice

**DOI:** 10.1371/journal.pone.0056636

**Published:** 2013-02-25

**Authors:** Francesca Farina, Giulio Sancini, Cristina Battaglia, Valentina Tinaglia, Paride Mantecca, Marina Camatini, Paola Palestini

**Affiliations:** 1 Department of Health Science, POLARIS Research Center, University of Milano-Bicocca, Monza, Italy; 2 Department of Medical Biotechnologies and Translational Medicine (BIOMETRA), PhD School of Molecular Medicine, University of Milano, Segrate, Italy; 3 Department of Environmental Science, POLARIS Research Center, University of Milano-Bicocca, Milano, Italy; Virginia Commonwealth University, United States of America

## Abstract

Recent studies have suggested a link between particulate matter (PM) exposure and increased mortality and morbidity associated with pulmonary and cardiovascular diseases; accumulating evidences point to a new role for air pollution in CNS diseases. The purpose of our study is to investigate PM10sum effects on lungs and extra pulmonary tissues. Milano PM10sum has been intratracheally instilled into BALB/c mice. Broncho Alveolar Lavage fluid, lung parenchyma, heart and brain were screened for markers of inflammation (cell counts, cytokines, ET-1, HO-1, MPO, iNOS), cytotoxicity (LDH, ALP, Hsp70, Caspase8-p18, Caspase3-p17) for a putative pro-carcinogenic marker (Cyp1B1) and for TLR4 pathway activation. Brain was also investigated for CD68, TNF-α, GFAP. In blood, cell counts were performed while plasma was screened for endothelial activation (sP-selectin, ET-1) and for inflammation markers (TNF-α, MIP-2, IL-1β, MPO). Genes up-regulation (HMOX1, Cyp1B1, IL-1β, MIP-2, MPO) and miR-21 have been investigated in lungs and blood. Inflammation in the respiratory tract of PM10sum-treated mice has been confirmed in BALf and lung parenchyma by increased PMNs percentage, increased ET-1, MPO and cytokines levels. A systemic spreading of lung inflammation in PM10sum-treated mice has been related to the increased blood total cell count and neutrophils percentage, as well as to increased blood MPO. The blood-endothelium interface activation has been confirmed by significant increases of plasma ET-1 and sP-selectin. Furthermore PM10sum induced heart endothelial activation and PAHs metabolism, proved by increased ET-1 and Cyp1B1 levels. Moreover, PM10sum causes an increase in brain HO-1 and ET-1. These results state the translocation of inflammation mediators, ultrafine particles, LPS, metals associated to PM10sum, from lungs to bloodstream, thus triggering a systemic reaction, mainly involving heart and brain. Our results provided additional insight into the toxicity of PM10sum and could facilitate shedding light on mechanisms underlying the development of urban air pollution related diseases.

## Introduction

Short term exposure to a high amount of PM10 (particles ≤10 µm in aerodynamic diameter, including fine and ultrafine particles) leads to higher hospitalization rates for cardiovascular diseases, increases risk for myocardial infarction and ischemic stroke [1], even 24 h after peak of pollution [2]. Different possible mechanisms have been hypothesized to explain the systemic effect of PM inhalation [3], [4]: on one hand, the inhaled PM may trigger the direct release of pro-oxidative and/or pro-inflammatory mediators from lungs into systemic circulation and, on the other hand, ultrafine PM may actively translocate from lungs into the bloodstream thus exerting extra pulmonary toxicity.

Convincing evidences indicate that PM10 causes the most severe effects on human health because of the broad range of miscellaneous toxic compounds present in this PM fraction [1], [3], such as transition metals, endotoxins [5] and ultrafine components, which could mediate the adverse effects in a mechanism called the “ultrafine hypothesis” [6].

Brain is another potential target of the inhaled PM: it is known that inhaled nanosized particles can penetrate lungs, deposit in extra pulmonary tissues and cross the blood-brain barrier (BBB), possibly already compromised by the PM triggered systemic inflammation [7].

Our previous investigations [8] demonstrated that summer PM10 (PM10sum) induced more severe lung inflammation than winter PM10 (PM10win) after a single intratracheal instillation in mice, and suggested a link between pro-inflammogenic potential of PM10sum and its Gram-negative bacteria content [9]. The greater LPS amount associated to PM10sum (60,5 EU/mg) [10] could induce in lungs a condition that might trigger a systemic toxic reaction, so that the aim of the present study is to broaden the analysis of Milano PM10sum-induced toxicity, not only in the respiratory tract but also in extra pulmonary districts. To disclose the systemic toxicity of Milano PM10sum, we here analysed different pro-inflammatory and cytotoxic markers, both at RNA as well as at protein level, in BALB/c mice broncho-alveolar lavage fluid (BALf), lungs, blood, heart and brain, 24 hours after the last of three intratracheal instillations of PM10sum. Finally we evaluated the microRNA miR-21, recently proposed as a new marker of inflammation which is up-regulated by LPS in many cells types, including macrophages [11].

## Materials and Methods

### Animals

Male BALB/c mice (7–8 weeks old) were purchased from Harlan; food and water were administered *ad libitum.* Mice were housed in plastic cages under controlled environmental conditions (temperature 19–21°C, humidity 40–70%, lights on 7 a.m.−7 p.m.). “Animal use and care procedures were approved by the Institutional Animal Care and Use Committee of the University of Milano-Bicocca and complied with guidelines set by Italian Ministry of Health (DL 116/92); invasive procedures have been performed under anesthesia and all efforts were made to minimize suffering.”

### PM Sources and Characterization

Atmospheric PM10sum was collected during summer 2008 at Torre Sarca, an urban site in Milano, as described in previous papers [12]. The chemical characterization of PM10 collected during summer 2008 doesn’t differ from the PM10 collected in summer 2006 and 2007 [10]. Particles were recovered from filters by sequential sonications (four cycles of 20 min each) in sterile water; detached particles were dried into a desiccator and weighed. Particles’ suspensions were prepared as follow: just before the intratracheal instillation, PM10sum aliquots were properly diluted in sterile saline, sonicated, vortexed and then immediately instilled in mice.

### Dose

A single intratracheal instillation of 100 µg of PM10sum raised pulmonary inflammation within 3 hours; 24 h after the single instillation all the inflammatory markers basically turned to sham levels, and only differential cell count and Hsp70 reverted 1 week later [8].

The aim of this study is to disclose the pulmonary short-term effects and extra-pulmonary translocation of PM10sum collected in Milano urban centre. Similar investigations have been previously based on very high PM exposure rate both in case of whole chamber PM exposure [13], [14], [15], [16], [17], [18], [19] and in single or repeated intratracheal instillations [20], [21], [22], [23], [24], [25].

It is well known that larger particles in the lungs are rapidly phagocytised by AMs while smaller particles enter the blood capillaries [20]; moreover, pulmonary inflammation plays a key role in enhancing the extra-pulmonary translocation of particles, as confirmed by the evidence that particles translocation is markedly increased following LPS treatment [20]. We have previously reported that LPS concentration is particularly high in PM10sum collected in Milano (60 EU/mg) [10] and that PM10sum sub-fraction contains about 60% of fine and ultrafine particles [26]. Our treatment scheme has been designed to lengthen the PM10sum triggered pro-inflammatory effects within lungs, in order to prove the translocation of inflammatory mediators, cytokines, ultrafine particles, LPS and/or PM associated metals from lungs toward the bloodstream. We started from the PM dose proposed by Happo et al. [24], who instilled in mice a cumulative dose of 0.82 mg/animal of coarse PM, and we reduced the cumulative dose to 0.3 mg/animal of PM10sum within the same time points. Indeed, the PM dose here used is not directly correlated to human urban exposures, but it has been determined as the lowest dose which induces a sustained lung inflammatory response in PM10sum exposed mice.

It must be taken into account that not all the particles intratracheally instilled are able to reach the alveoli, as some are quickly removed by the muco-ciliar clearance system or phagocited by alveolar macrophages. Finally, we used healthy BALB/c mice, preventing additional variability to response due to pre-existing disease. In any case, no signs of PM10sum lung overloading have been reported as clearly demonstrated by histological and biochemical investigations.

### Intratracheal PM10sum Instillation

Animal testing was carried out by intratracheally instilling 3 mice for each experimental group and the experiment was replicated twice, for a total of 6 sham and 6 PM10sum-treated mice. For RNA and miRNA analysis, additional 5 sham and 5 PM10sum-treated mice were considered.

Male BALB/c mice were briefly exposed to a mixture of 2.5% isoflurane (Flurane) anesthetic gas and kept under anaesthesia for the whole instillation procedure (about 5 minutes). Intratracheal instillation with 100 µg of PM10sum in 100 µl of isotonic saline solution or 100 µl of isotonic saline solution (sham) has been achieved by means of MicroSprayer® Aerosolizer system (MicroSprayer® Aerosolizer- Model IA-1C and FMJ-250 High Pressure Syringe, Penn Century, USA; validated by Bivas-Benita [27], as described in Mantecca et al. [28], [29] and in Farina et al. [8]. The intratracheal instillation was performed on days 0, 3, and 6, for a total of three instillations, as previously described [24], [25].

### Bronchoalveolar Lavage Fluid Analysis

24 h after the last instillation, mice from each experimental group (sham and PM10sum-treated) were euthanized with an anesthetic mixture overdose (Tiletamine/Zolazepam-Xylazine and Flurane). The PM adverse effects were assessed 24 h after the last treatment of repeated dosing of particulate since the greatest and prolonged inflammation does occur within this time point [24]. The Broncho Alveolar Lavage Fluid (BALf), pellets and supernatants have been collected as described in Mantecca et al. [28], [29] and in Farina et al. [8].

#### Cell counts

After centrifugation, total and differential cell counts were performed according to Mantecca et al. [28], [29] and Farina et al. [8].

#### Cytokines analysis

The analysis of pro-inflammatory cytokines and chemokines released in the BALf was performed by DuoSet ELISA kits for TNF-α, MIP-2 and IL-1β(R&D Systems, Minneapolis, MN) according to the manufacturer’s protocols.

#### Biochemical analysis

The following biochemical analysis were performed on cell-free BALf supernatants. The commercially available kits for ALP (DALP-250 QuantiChrom Alkaline Phosphatase Assay Kit, Gentaur Molecular) and LDH (DLDH-100 QuantiChrom Lactate Dehydrogenase Kit, Gentaur Molecular) were employed according to the manufacturer’s instructions.

#### Other proteins

30 µg of BALf proteins of sham and PM10sum-treated mice were loaded on SDS-PAGE, submitted to electrophoresis followed by Western blot, and tested for MPO and Hsp70 (anti-MPO 1∶200, anti-Hsp70 1:200, Santa Cruz), according to the procedures below described.

### Lung, Heart and Brain Parenchyma Analysis

Lungs of sham and PM10sum-treated mice were quickly excised from the chest and washed in ice cold isotonic saline solution. Left lung from sham and PM10sum-treated mice was dissected and submitted to histological and immunohystochemical analysis, while right lung was submitted to immunoblotting analysis. For proteins detection and quantification, organs were minced at 4°C, briefly homogenized for 30 seconds at 11000 rpm with Ultra-Turrax T25 basic (IKA WERKE), sonicated for 30 seconds and then suspended in NaCl 0.9%. Samples were submitted to trichloroacetic acid (TCA) precipitation according to the procedure described in Farina et al. [8]. The pellets were suspended in water and protein amounts measured by BCA method (Sigma Aldrich, USA).

Thereafter, lung, heart and brain homogenates of sham and PM10sum-treated mice were loaded on SDS-PAGE and submitted to electrophoresis, followed by Western blot, according to the procedures described in Farina et al. [8]. Lung parenchyma was assessed with specific antibodies for MPO, ET-1, HO-1, Cyp1B1, iNOS, Hsp70, Caspase8-p18 and Caspase3-p17 (all 1∶200, Santa Cruz). The activation of TLR4 pathway has been studied by investigating the phosphorylation of IRAK-1, TAK1 and IKBα (all 1∶200, Abcam). Heart homogenates were incubated with specific antibodies for MPO, HO-1, Hsp70, Cyp1B1, ET-1 Caspase8-p18 and Caspase3-p17 (all 1∶200, Santa Cruz) and brain homogenates were evaluated with specific antibodies for ET-1, HO-1, soluble and membrane bound TNF-α, Hsp70, Cyp1B1, CD68, GFAP, Caspase8-p18 and Caspase3-p17 (all 1∶200, Santa Cruz).

Then, blots were incubated for 1.5 h with horseradish peroxidase-conjugated anti-rabbit IgG (1∶5000) or anti-goat IgG (1∶2000) diluted in PBS-Tween20/milk or in TBS-Tween20/BSA. Proteins were detected by ECL by means of the SuperSignal detection kit (Pierce, Rockford, IL). Immunoblot bands were analyzed and the optical density (OD) quantified by KODAK (Kodak Image Station 2000R); all the data have been normalized to β-actin (1∶1500, Sigma) and each protein in PM10-treated group has been normalized to the respective sham group.

### Lung Histological Analysis

At the end of BAL procedure, the left lung from sham and PM10sum-treated mice was excised and immediately formalin fixed and processed for histology. Briefly, after being preserved for 24 h in the fixative, tissue samples were rinsed in distilled water, dehydrated in an ethanol series from 70% to 100% and embedded in Bio-plast tissue embedding medium. For each sham and PM10sum exposed lung sample, 7.0 µm serial sections were cut by a rotary microtome, mounted on slides and stained with Mayer’s haemalaun and alcoholic eosin.

The immunohistochemical localization of HO-1 in lung tissues was performed by an indirect immunochemical method using a rabbit anti-HO-1 polyclonal antibody (Santa Cruz), and the peroxidase-based Vectastain Elite ABC Kit (Vectastain Laboratories) following the procedure reported in Mantecca et al. [29].

Samples were qualitatively screened by means of Zeiss Axioplan microscope at a magnification of 40× and images were taken using Zeiss AxioCam MRc5 digital camera interfaced with the Axiovision Real 4.6 software. Figure panels were prepared using Adobe Photoshop.

### Blood Analysis

Blood of sham and PM10sum-treated mice was collected by means of intracardiac puncture with the appropriate anticoagulant (EDTA, Na-citrate). Total cells count and neutrophils percentage were performed as previously described [8]. Plasma was obtained after two centrifugation, the first at 2000 g for 20 minutes and the second at 10000 g for 10 minutes at 4°C to completely remove platelets. Plasma samples have been then submitted to sP-Selectin quantification (Quantikine Mouse sP-selectin, R&D Systems) and cytokines analysis (TNF-α, MIP-2 and IL-1β; R&D Systems, Minneapolis, MN), following manufacturer’s procedures. Immunoblot assays of MPO and ET-1 (all 1∶200, Santa Cruz) were performed according to previously described protocols; all the data have been normalized to albumin. Each protein of PM10sum-treated group has been normalized to the respective sham group.

### RNA e miRNA Expression Analysis

5 sham and 5 PM10sum-treated mice were considered for RNA analysis. Lungs, not submitted to BAL procedure (called “no-BAL”), have been excised, suspended in an appropriate volume of RNA Later and submitted to total RNA extraction. Aliquots of lung and heart parenchyma from all the 5 sham and 5 PM10sum-treated mice were submitted to immunoblot analysis. Total RNA was extracted from tissues using the miRNeasy extraction kit (Qiagen, Hilden, Germany), according to the manufacturer’s instructions. Blood sample collection and RNA extraction were carried out using the Mouse RiboPure™-Blood RNA Isolation kit (Ambion, Life Technologies, Carlsbad, CA, USA), following the manufacturer’s instructions. After elution in RNase free-water, RNA samples were quantified by ND-1000 spectrophotometer (NanoDrop Technologies, Wilmington, DE, USA). RNA quality was checked by microcapillary electrophoresis with 2100 BioAnalyzer (Agilent Technologies, Santa Clara, CA, USA). Total RNA integrity was assessed on the basis of the RIN (RNA Integrity Number) factor and presence of low molecular weight RNA molecules (including 5S rRNA and small RNAs) was verified. RNA samples were stored at −80°C until use.

Quantitative PCR (QPCR) reactions for microRNAs was performed by use of TaqMan® MicroRNA Reverse Transcription (RT) kit (Applied Biosystems, Life Technologies, Inc. Carlsbad, CA, USA) and of specific miRNA primers provided with TaqMan® microRNA assays, according to the manufacturer’s protocol. Starting from 10 ng of total RNA for each assay, RT reactions were performed by means of Applied Biosystems 7900 Thermocycler machine. Quantitative microRNA expression analysis was carried out for miR-21 (Assay ID, 000397, Applied Biosystems) normalized against U6snRNA (Assay ID, 001973, Applied Biosystems) taken as endogenous control. For gene expression analysis, we performed QPCR starting from 1 µg of total RNA using the High Capacity cDNA Reverse Transcription kit (Applied Biosystems) and gene-specific primers provided with TaqMan® Gene Expression Assays. Specifically, QPCR analysis were carried out for HMOX1 (Assay ID Mm00516005_m1), Cyp1B1 (Assay ID Mm00487229_m1), IL-1β (Assay ID Mm01336189_m1), MIP-2 (Assay ID Mm00436450_m1) and MPO (Assay ID Mm00447886_m1) genes. All data have been normalized versus glyceraldehyde*-*3*-*phosphate dehydrogenase (GAPDH, Assay ID Mm99999915_g1) gene taken as endogenous control. Reactions were run in triplicate on the Applied Biosystems 7900HT Fast Real-Time PCR System machine. Ct values were calculated using the SDS software version 2.3 (Applied Biosystems), by applying automatic baseline and standard threshold settings. We applied the 2^−ΔΔCt^ method (Applied Biosystems User Bulletin No. 2) to obtain a relative quantification of miRNA and gene expression levels.

### Statistical Analysis

For each cytological and biochemical parameter measured in sham and PM10sum treated mice the means ± standard error (s.e.) were calculated. Statistical differences were confirmed by U Mann-Withney test. To test the tendency toward increase or decrease of the cytological and biochemical parameters, linear regressions were carried out. Statistical differences were considered to be significant at the 95% or 99% level (p<0.05 or p<0.01).

## Results

### BALf Analysis

**Figure 1:**
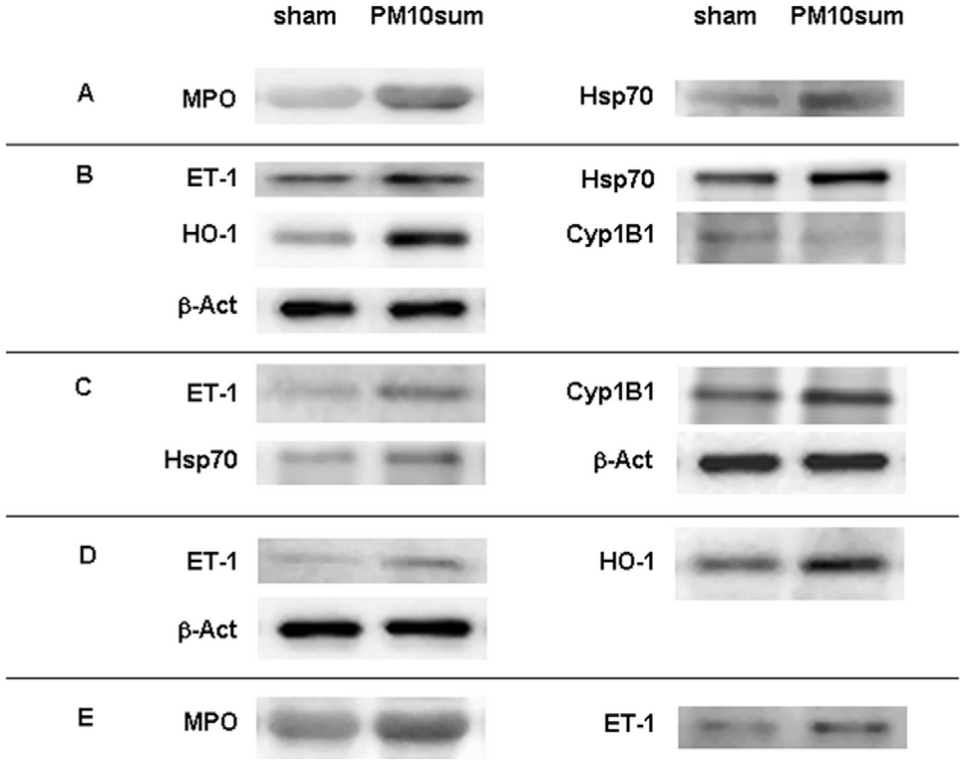
BALf, lung, heart, brain and plasma immunoblotings. Western blottings displaying proteins in BALf (A), lung (B), heart (C), brain (D) and plasma (E) in sham and PM10sum-treated mice, 24 h after the third intratracheal instillation. Reported blots are representative of 6 sham and 6 PM10sum-treated mice; densitometry analyses are reported in Tab.1.

Total cell count confirmed no differences between sham and PM10sum-treated mice, 24 h after the last PM10sum intratracheal instillation (Tab. 1, A). On the contrary, differential cell count disclosed a significant decrease of AMs percentage and a significant increase of PMNs percentage (99% neutrophils) in treated mice (Tab. 1, A). A significant increase of IL-1β and MIP-2 concentrations were evident in the BALf of PM10sum-treated mice in comparison to sham, though in presence of unchanged concentration of TNF-α (Tab. 1, A). Lactate Dehydrogenase (LDH) and Alkaline Phosphatase (ALP) activities were analyzed in the BALf as markers of cytotoxicity. LDH activity was significantly increased in PM10sum-treated mice (Tab. 1, A), while no difference was detected in ALP activity (Tab. 1, A). Finally, PM10sum-treatment induced a significant increase in both MPO and Hsp70 levels (Fig. 1, A and Tab. 1, B).

**Table 1:** BALf analysis.

BALf		sham (n = 6)		PM10sum (n = 6)		
		mean	± s.e.	mean	± s.e.	p
**A**	Total cells (E+06)	5.21	1.34	5.93	2.31	
	AMs%	70.2	9.48	41.33	3.73	**
	PMNs%	28.8	9.91	57.91	4.02	*
	Ls%	0.97	0.97	0.68	0.68	
	TNF-α (pg/mL)	157.7	32.21	310.98	94.5	
	MIP-2 (pg/mL)	49.46	33.9	271.09	64.11	*
	IL-1β (pg/mL)	40.63	17.46	164.35	36.39	*
	LDH (IU/L)	30.7	2.46	43.43	4.46	*
	ALP (IU/L)	0.37	0.11	0.36	0.07	
**B**	MPO	1	0.23	2.91	0.55	*
	Hsp70	1	0.31	3.19	0.72	*

Several markers have been analysed in the BALf of sham and PM10sum-treated mice. (A): table summarizing results of cell counts and biochemical analysis in BALf from sham (n = 6) and PM10sum-treated mice (n = 6), 24 h after the third intratracheal instillation; (B): immunoblotting results in BALf from sham (n = 6) and PM10sum-treated mice (n = 6), 24 h after the third intratracheal instillation; each protein in PM10-treated group has been normalized onto respective sham group. All the data are expressed as mean ± s.e. Sham *vs* PM10sum-treated:

* p<0.05,

** p<0.01.

### Lung Genes Expression Analysis

PM10sum-treatment induced a significant MIP-2 up-regulation while no significant changes were found for HMOX1, Cyp1B1, IL-1β and MPO; an induction of miR-21 in the lung tissues of PM10sum-treated mice was evidenced during miRNA gene expression analysis (Tab. 2).

**Table 2:** Lung gene expression.

LUNG	fold increase	range	± s.e.	p
HMOX1	1.51	0.68–2.10	0.39	
Cyp1B1	1.29	1.03–1.71	0.24	
IL-1β	1.07	0.93–1.32	0.16	
MIP-2	2.55	2.03–2.90	0.15	*
MPO	2.21	1.21–3.02	0.4	
miR-21	3.04	1.47–5.55	0.59	*

QPCR gene expression analysis in lung parenchyma from sham (n = 5) and PM10sum-treated mice (n = 5), 24 h after the third intratracheal instillation. Sham vs. PM10sum-treated:

* p<0.05.

### Lung, Heart and Brain Protein Analyses

ET-1, a potent regulator of the vascular tone, was significantly higher in the lungs of PM10sum-treated mice in comparison to sham (Fig. 1, B and Tab. 3, A). Moreover a significant increase in HO-1 and Hsp70 levels (Fig. 1, B and Tab. 3, A), both involved in the protection against protein unfolding, as well as in the inflammation and oxidative stress, were observed in the lungs of PM10sum-treated mice. The level of Cyp1B1, a cytochrome of the P450 superfamily involved in the activation of many xenobiotics and in PAHs metabolism, significantly decreased 24 h after the last PM10sum intratracheal instillation (Fig. 1, B and Tab. 3, A). iNOS, MPO, Caspase8-p18 and Caspase3-p17 showed no differences between sham and PM10sum-treated mice (Tab. 3, A). TLR4 pathway was not activated as no increases of the IRAK1, TAK1 and IKBα phosphorylation were detected (Tab. 3, A).

**Table 3:** Immunoblotting analysis.

		sham (n = 6)		PM10sum (n = 6)		
		mean	± s.e.	mean	± s.e.	p
**A- LUNG**	ET-1	1	0.1	1.69	0.52	*
	HO-1	1	0.14	3.07	0.64	*
	Hsp70	1	0.19	1.68	0.23	*
	Cyp1B1	1	0.26	0.39	0.11	*
	iNOS	1	0.14	0.75	0.08	
	MPO	1	0.24	1.32	0.32	
	Casp8-p18	1	0.24	1.04	0.15	
	Casp3-p17	1	0.32	1.1	0.2	
	pIRAK1/IRAK1	1	0.16	0.66	0.09	
	pTAK1/TAK1	1	0.24	1.21	0.12	
	pIKBα/IKBα	1	0.18	1.05	0.08	
**B- HEART**	ET-1	1	0.24	2.41	0.58	*
	Cyp1B1	1	0.37	2.65	0.18	*
	Hsp70	1	0.33	2.04	0.11	*
	HO-1	1	0.44	1.5	0.27	
	MPO	1	0.26	2.45	0.11	
	Casp8-p18	1	0.27	1.02	0.14	
	Casp3-p17	1	0.34	0.72	0.22	
**C- BRAIN**	ET-1	1	0.2	2.86	0.86	*
	HO-1	1	0.22	2.3	0.72	*
	Cyp1B1	1	0.31	0.68	0.25	
	CD68	1	1.14	4.5	1.06	
	soluble TNF-α	1	0.24	2.22	0.36	
	memb TNF-α	1	0.2	1.19	0.41	
	Hsp70	1	0.2	1.17	0.28	
	GFAP	1	0.29	1.03	0.22	
	Casp8-p18	1	0.31	1.35	0.52	
	Casp3-p17	1	0.29	1.65	0.42	

Immunoblotting analysis in lung (A), heart (B) and brain (C) parenchyma from sham (n = 6) and PM10sum-treated mice (n = 6), 24 h after the third intratracheal instillation. The proteins have been normalized to β-actin and each protein in PM10-treated group has been normalized onto respective sham group. All the data are expressed as mean ± s.e. Sham vs. PM10sum-treated:

* p<0.05.

Heart tissues of PM10sum-treated mice disclosed significant increases of ET-1, Cyp1B1 and Hsp70 levels (Fig. 1, C and Tab. 3, B). MPO, HO-1, Caspase8-p18 and Caspase3-p17 shown no differences between sham and PM10sum treated mice (Tab. 3, B).

In the brain of PM10sum-treated mice, ET-1 and HO-1 levels were significantly increased (Fig. 1, D and Tab. 3, C); Cyp1B1, CD68, soluble and membrane bound TNF-α, Hsp-70, GFAP, Caspase8-p18, Caspase3-p17 were basically unchanged comparing sham and PM10sum-treated mice (Tab. 3, C).

### Blood and Plasma Analysis

Gene expression analysis evidenced no up-regulation of HMOX1 or IL-1β in blood RNA of PM10sum-treated mice, compared to sham, together with no induction of miR-21 (Tab. 4).

**Table 4:** Blood gene expression.

BLOOD	fold increase	range	± s.e.	p
HMOX1	2.21	1.00–5.37	0.95	
IL-1β	1.23	0.52–2.39	0.66	
miR-21	1.38	0.82–1.96	0.43	

QPCR gene expression analysis in blood from sham (n = 5) and PM10sum-treated mice (n = 5), 24 h after the third intratracheal instillation.

Blood and plasma of sham and PM10sum-treated mice were analysed for pro-inflammatory and pro-trombogenic markers. Total cell count, neutrophils percentage and sP-selectin concentration, a marker of the activated platelet/endothelium interface, were significantly increased 24 h after the last intratracheal instillation of PM10sum (Tab. 5, A). The cytokines analyses in the plasma of sham and PM10sum-trated mice, performed by ELISA assay, were under the kit detection limits.

**Table 5:** Blood/plasma analysis.

BLOOD		sham (n = 6)		PM10sum (n = 6)		
		mean	± s.e.	mean	± s.e.	p
**A**	Total cells (cells/mm3)	4275	218.2	5718	454.91	*
	Neutrophils (%)	5.3	0.86	14.9	1.37	**
	sP-selectin (ng/mL)	78.12	3.98	97.98	3.02	*
	TNF-α (pg/mL)	n.d.		n.d.		
	MIP-2 (pg/mL)	n.d.		n.d.		
	IL-1β (pg/mL)	n.d.		n.d.		
**B**	MPO	1	0.07	1.36	0.06	*
	ET-1	1	0.15	2.18	0.35	*

Inflammation and coagulation markers have been analysed in blood/plasma of sham and PM10sum-treated mice. (A): table summarizing assays in blood/plasma from sham (n = 6) and PM10sum-treated mice (n = 6), 24 h after the third intratracheal instillation; (B): immunoblotting results in plasma from sham (n = 6) and PM10sum-treated mice (n = 6), 24 h after the third intratracheal instillation. The proteins have been normalized to albumin and each protein in PM10-treated group has been normalized onto respective sham group. All the data are expressed as mean ± s.e. Sham vs. PM10sum-treated:

* p<0.05;

** p<0.01.

Finally, both MPO and ET-1 increased in PM10sum-treated mice (Fig. 1, E and Tab. 5, B).

### Histopathological Findings

Despite the relatively high cumulative dose of PM10sum instilled, there were no signs of pulmonary overloading with particles; moreover, only few free particles and no large particle aggregates were seen in the BALf or lung tissue of treated mice.

**Figure 2:**
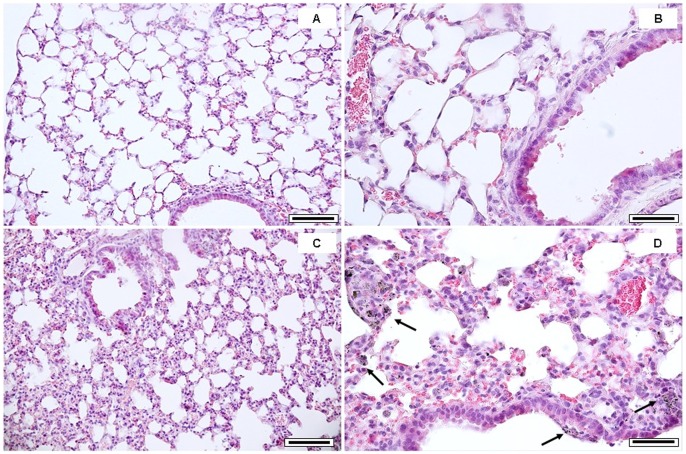
Lung histological analysis. Histology of lung tissue 24 h after the third intratracheal instillation. (A, B): lung parenchyma of sham mice, showing bronchiolar and alveolar epithelia; (C, D): PM10sum-treated lung parenchyma, showing inflammatory cells recruitment and AMs infiltration (arrows) in the connective surrounding terminal bronchioles and proximal alveolar sacs. Each figure represents the status evidenced examining 6 sham and 6 PM10sum-treated mice. A and C bars = 150 µm; B and D bars = 50 µm.

**Figure 3:**
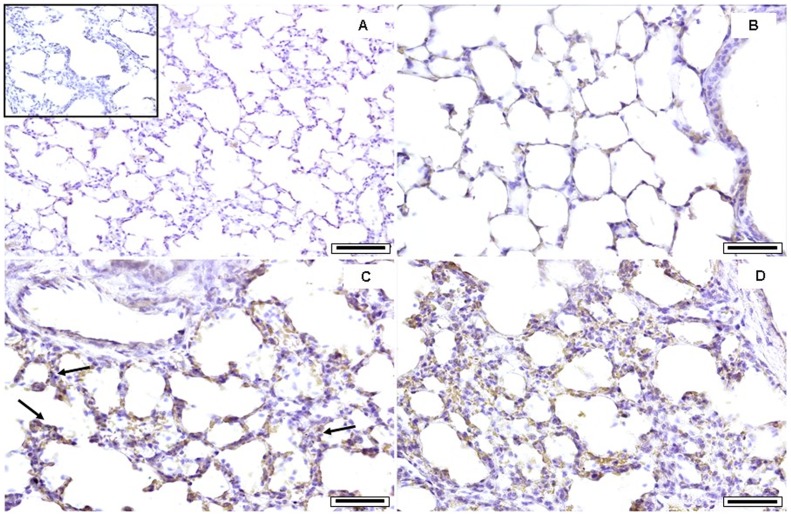
Lung immunohistochemical analysis. Immunohistochemical analyses of HO-1 expression in lung tissue 24 h after the third intratracheal instillation; the brown color reflects the site and intensity of HO-1 expression. (A): lung of sham mice; in the box, a control of the immunohistochemical reaction specificity, where the primary antibody has been avoided, in a PM10sum instilled mouse; (B): alveolar and peribronchiolar spaces in the lung of a sham mouse; (C, D): alveolar and peribronchiolar spaces in lungs of PM10sum-treated mice. Each figure represents the status evidenced examining 6 sham and 6 PM10sum-treated mice. A bar = 150 µm; B–D bars = 50 µm.

As evidenced by the histological lung analysis, when compared to sham (Fig. 2, A–B) PM10sum has induced inflammatory cell recruitment in the connective surrounding the terminal bronchioles and the proximal alveolar sacs (Fig. 2, C–D). Lungs instilled with PM10sum showed extended inflammatory infiltrate and particles engulfed by alveolar macrophages (Fig. 2, C–D). These histological evaluations have supported the cytological and biochemical assays on BALf. Immunohistochemical detection of HO-1 in the lungs of PM10sum-treated mice has confirmed the biochemical analyses, (Fig. 3, A–D). HO-1 induction appeared to be distributed along both airways and the respiratory tract, particularly well distinguishable within the epithelium of distal bronchioles (Fig. 3, C–D).

## Discussion

Prolonged or persistent exposure to PM is supposed to exert heavy adverse effects on cell homeostasis mediated by a direct particles cell interaction within the district that PM reaches (lung, myocardial or neuronal tissues), or by the induction of a chronic inflammation, resulting in a general systemic inflammation and sustained oxidative stress status [30].

In order to enhance the translocation of PM10sum we tested the threshold able to lengthen the PM10sum pro-inflammatory effects within lungs of our BALB/c mice. Previous published reports of particles exposures that do not yield significant alterations in markers of lung inflammation [31], [32], but do show significant changes in the expression of makers of inflammation and endothelial activation in extra-pulmonary tissues, are basically related to engine combustion derived particles. Indeed the translocation of these fine particles might be quick enough to directly exert systemic adverse effects even without overt pulmonary effects.

### Inflammatory Response Elicited by PM10sum in Respiratory and Cardiovascular Systems

Lungs are the primary site of exposure to PM. In the BALf from healthy mice, the alveolar macrophages are the most numerous cells (>90%) and an influx of neutrophils is a sensitive indicator of inflammatory reaction [28], [29], [33]. After three PM10sum intratracheal instillations, differential cell count evidenced a significant decrease of AMs percentage and a significant increase of PMNs percentage. A compensatory effect between the infiltration of PMNs and the extra-pulmonary migration of AMs is supposed to take place, thus explaining the absence of difference of the total cell count between sham and PM10sum-treated mice, as previously described [8].

Comparing to a single exposition [8], three repeated intratracheal instillations of PM10sum showed a lower PMNs percentage and a higher AMs percentage in the BALf of treated mice, confirming that the acute inflammatory status is still sustained but not heavily exacerbated, considering that the lung clearance mechanisms are still working.

24 h after a single PM10sum instillation, TNF-α concentration significantly increased in the BALf of treated mice [8]; nevertheless, 24 h after the last of three PM10sum instillations in a week (cumulative dose of 0.3 mg/mice/week), TNF-α concentration was basically unchanged comparing to sham. Moreover, histological findings have not shown signs of lung particle overloading. All together these observations claim for a mild ongoing lung inflammation triggered by PM10sum treatment scheme. This perfectly agree with the data coming from the extra-pulmonary translocation of lung administered particles both in humans and rodents [14], [15], [16], [17], [19], [20], [22], [23].

Several chemoattractants derived from macrophages and epithelial cells might be responsible for neutrophils recruitment in the lung, in particular IL-1β [34]. 24 h after PM10sum treatment, the significant increase of IL-1β concentration in the BALf together with neutrophils infiltration suggests an ongoing and persistent lung inflammation [35]. Indeed, the finding of increased MIP-2 lung mRNA and BALf MIP-2 concentration observed after 24 h in PM10sum-treated mice is in agreement with the quick induction in MIP-2 mRNA observed in rat lungs after *in-vivo* stimulation with IL-1β [34].

The increased MPO level in the BALf of PM10sum-treated mice perfectly correlates with lung neutrophils infiltration: indeed, MPO is an inflammatory marker released by degranulation of activated neutrophils promoted by IL-1β [36], [37] and its activity increases after PM exposure [38].

The pro-inflammatory effect of PM with high LPS amount on lungs of treated mice has been already described [39]. It is known that mediators produced in the lungs are able to translocate into the bloodstream [40] thus we analysed the presence of inflammatory markers also in blood and heart tissue of sham and PM10sum-treated mice.

The lack of increase in circulating cytokines after acute particles exposure might reflect that the PM induced inflammation is still localized (i.e. confined to the lungs); however, after repeated PM-exposure, the inflammatory cytokines released from lungs may have enough time to enter the systemic circulation and become detectable [41]. Indeed, the increased total cell number, neutrophilia and high MPO levels, that we found in blood and plasma of PM10sum-treated mice, support the hypothesis of a systemic reaction and confirm the huge pro-inflammatory effect of PM10sum.

### Endothelial Activation in Lungs and Cardiovascular System

An increase of vasoactive mediators may occur within the lungs and the systemic circulation both in presence or absence of lung inflammation [38]. Previous studies have shown that PM exposure is associated with an increases in ET-1 level *in-vivo* and *in-vitro* [42], [43].

In the lung parenchyma, the ET-1 expression in macrophages as well as in endothelial and epithelial cells is stimulated by cytokines, LPS, air pollutant and ozone [44], [45]; ET-1 could promote acute lung injury, inducing cytokines production and activating neutrophils, releasing oxygen radicals [44]. Indeed, the increased ET-1, that we found in the lungs of PM10sum-treated mice, once more confirms an ongoing lung inflammation linked to PM10sum exposure.

In the heart, ET-1 is synthesized by cardiomyocytes, fibroblasts, and endothelial cells; moreover, ET-1 directly stimulates cardiac fibroblasts to produce extracellular matrix proteins thus promoting myocardial fibrosis [46]. The ET-1 expression has increased in cardiac tissue of rats daily exposed to diesel exhaust particles and to urban particulate matter [47]. Confirming previous findings, the high ET-1 level observed in the heart of our PM10sum-treated mice strengthens the evidences of cardiac adverse effects elicited by PM10sum exposure.

A significant increase of ET-1 and sP-selectin were observed in plasma of PM10sum-treated mice. As previously discussed, lung cells may release ET-1 into the systemic circulation after PM exposure, as well as the vascular ET system may be up-regulated after PM exposure [38], [48]. Increased levels of circulating ET-1 may trigger arterial vasoconstriction and influence the inflammatory response by promoting the recruitment of leukocytes into vessel walls [49]. High ET-1 within the plasma correlates with acute coronary risk in concomitance to PM exposure [50].

Many data indicate that platelet adhesion to the endothelium is an event preceding the development of atherosclerotic lesions [51], contributing to the final stages of cardiovascular diseases [52]. sP-selectin is a marker of activated interface blood-vasculature [53]; the significant increase in sP-selectin plasma concentration of PM10sum-treated mice suggested a systemic endothelial activation induced by PM10sum, which might be related to the high rate of cardiovascular hospitalization following PM10 exposure peak.

Finally, high MPO levels that we found in blood of PM10sum-treated mice could be related to an heavy endothelial dysfunction, as discussed by Brook et al. [38]. The increase of MPO could be potentially harmful, due to the generation of hypochlorous acid, a compound toxic on bacteria and also onto host cells [37].

### PM10sum Cytotoxic Effect in Lungs and Heart

A significant increase of LDH activity in the BALf was observed 24 h after the PM10sum third intratracheal instillation. However, this evidence was not supported by any changes in ALP activity, a specific marker of damage of type I pneumocytes and of proliferation of type II pneumocytes [54]. The direct cytotoxic damage induced by PM10sum might be basically related to AMs and only marginally to pneumocytes, as previously we described for winter PM [8]; it is possible to hypothesize that the damage produced by repeated PM intratracheal instillations is a consequence of the amounts of metals and PAHs still associated to PM10sum.

The lung parenchyma necrosis and inflammation could explain the increase of Hsp70 levels that we found in the BALf after PM10sum instillation. Hsp70 might increase in the extracellular milieu, where it acts as a cytokine to human monocytes [55], after two events: an active release from epithelial cells or from mononuclear cells and a passive release when cells undergo necrosis [56].

Finally, in both lungs and heart, 24 h after the last PM10sum-treatment, the initiator Caspase 8 and the effector Caspase 3 were not active, despite the presence of pro-apoptotic inducers such as cytokines and hypochlorous acid, eventually produced by MPO in the BALf and blood, suggesting the existence of an on-going protection mechanism.

### HO-1 and Hsp70 Induced Protection in Lung and Heart Parenchyma

HO-1 was significantly higher in the lungs of PM10sum-treated mice thus indicating the existence of a protection mechanism against oxidative stress and inflammation. In the lung parenchyma, HO-1 is expressed in various cells, including type II pneumocytes and AMs [57]. Its role is to catabolize the heme group from the cytosol, thus generating CO, biliverdin (converted to bilirubin) and Fe^2+^ thus playing a protective role against inflammation and oxidative stress [58]. Moreover, it has been hypothesized the existence of a post-translational down-regulation of cytochromes following the induction of HO-1, possibly related to a decrease in the heme group bioavailability [59]. Consistently with this observations, we found a significant decrease in Cyp1B1 levels in the lung parenchyma of PM10sum-treated mice.

On the contrary, the absence of a significant increase of HO-1 in the heart of PM10sum-treated mice allows the increase of Cyp1B1, thus suggesting that soluble molecules PM-related could reach the heart parenchyma through the bloodstream.

Oxidative stress, LPS, cytokines, ER stress and protein unfolding induced by PM exposure, might promote the expression of heat shock proteins, such as Hsp70 or Hsp90 [60]; in particular, the intracellular Hsp70 has the ability to protect cells and tissues limiting the amount of inflammatory mediators [61]. In both lung and heart parenchyma, Hsp70 showed a significant increase 24 h after the last PM10sum instillation, suggesting that the active protection system is not only related to the induction of HO-1. Graff et al. [62] demonstrated that the treatment of rat ventricular myocytes with Zn and V induced small but significant increases in the expression of Hsp70. Taking into account that Zn and V are soluble components PM-related and have the potential to be absorbed into the bloodstream and transported to the heart [62], [63], we hypothesize that these two metals could be in part accountable of the Hsp70 increase that we observed in PM10sum-treated mice.

Finally, both Hsp70 and HO-1, are important apoptotic inhibitors [64], [65] which might thus explaining the absence of apoptosis cascade activation that we found in lungs and heart parenchyma of PM10sum treated mice.

### miR-21

In order to advance our studies on the PM10sum toxic effects, we considered a new class of molecules, miRNAs. In particular, according to literature, miR-21 is one of the miRNAs induced in mice lungs by exposure to LPS [66] and is involved in negative regulation of the signalling pathway of TLR-2 [67] and TLR-4 [11], thus playing a key role in orchestrating the inflammatory process induced by LPS. Moschos et al. [66] and Sheedy et al. [11] have hypothesized that miR-21 might be involved in the resolution rather than in the induction of inflammation. So, increased miR21 levels that we found in the lungs of PM10sum-treated mice could be considered a marker of inflammatory status associated to an ongoing protection reaction. Consistent with this observation, the TLR4 pathway was not activated in the lungs of PM10sum-treated mice.

### PM10sum Toxicity on Brain

The BBB endothelial cells play an essential role in maintaining brain homeostasis, actively transporting nutrients, and limiting the entrance to harmful components such as LPS and cytokines [68]. In healthy brain, the endothelial cells express very low levels of adhesion molecules required for leukocyte migration [69], and expression of ET-1 increases BBB permeability *in-vivo* [68]. Guo et al. [35] proposed that elevated plasma levels of ET-1, induced by PM10 exposure *in-vivo*, may promote ET-1 penetration within the brain either by active transport or by passive diffusion across a permeabilized BBB. The high ET-1 levels that we found in the brains of PM10sum-treated mice suggest a compromised BBB function [7], therefore allowing the translocation of smallest particles and/or of their soluble components within brain. It has been disclosed that ET-1 is a key factor in the development of PM10-mediated brain endothelial injury.

A possible mechanism by which PM exerts its toxic effect on brain cells has been proposed by Block et al. [70]: it is supposed that particles might initiate the phagocytic activity of microglia, leading to the neurotoxic production of extracellular superoxide. As neurons are particularly vulnerable to oxidative damage, the ROS production can lead to neuron death. In our investigations, PM10sum-treatment causes an increase in brain HO-1 levels, probably as a consequence of oxidative stress induction.

The chronic induction of HO-1 can have both beneficial and detrimental effects on cellular metabolism. While exists plenty of evidence for the HO-1-mediated neuro-protection, a growing literature attesting the neuro-endangering effects of HO-1 activity is also at hand [71]. Fe^2+^ produced by HO-1 can catalyze the production of free radicals through the Fenton’s chemistry, thus acting as a cytotoxic and oxidative stress inducer. Indeed, the excessive cellular amounts of free Fe^2+^ heme-derived resulting from HO-1 over-activity and dysregulation of iron metabolism [72] may be related to the pathogenesis of neuro-degenerative disorders [73].

Finally, it must be noted that the intratracheal administration route for PM has completely avoided the particles transport along the olfactory nerve. Alterations in the nasal mucosa, olfactory bulb, cortical and sub-cortical brain structures have been described in healthy dogs exposed to high levels of ambient air pollutants [74]; in addition, brain biomarkers of oxidative stress and tissue injury have been observed in mice exposed to concentrated ambient particles [75]. Indeed, it is possible that ET-1 and HO-1 increased levels in the brain of our PM10sum-treated mice could be attenuated, while PM nasal inhalation could induce higher oxidative stress and endothelial activation in some regions of the central nervous system.

### Conclusions

A repeated exposure of PM10sum in BALB/c mice leads to the induction of lungs inflammation, associated to cytokine production, PMNs infiltration and endothelium activation. Translocation of mediators, cytokines, ultrafine particles, LPS and/or metals associated to PM10sum from lungs to bloodstream triggers systemic adverse effects, involving heart and brain. The activation of endothelium/platelets interface that we found in PM10 exposed mice could explain the associations between short-term induced changes by inhalable particles and cardiovascular hospital admissions. Our results confirm the systemic toxic effect triggered by PM10sum mainly involving the respiratory and cardiovascular system in an inflammatory and pro-coagulant reaction, thus intending a correlation between PM exposure and cardiovascular diseases. However, lungs and heart might activate some putative protection mechanisms against inflammation and/or cytotoxicity thus supporting that compensatory mechanisms may occur within sub acute PM exposure.

PM10sum promotes brain endothelial activation and oxidative stress induction. In recent years, increasing evidences indicates Alzheimer and other neurodegenerative diseases at least partially mediated by oxidative stress and impaired BBB functions.

These findings may contribute to the knowledge of the interplay between PM exposure and cardiovascular diseases, the chronic oxidative stress generation and the development of neurodegenerative diseases. Future investigations should address the effects of lifetime air pollution exposure and aging related to the neuroinflammation and to the neurotoxicity.
